# Supplementary material for: Detection, Identification and Molecular Characterization of the 16SrII-V Subgroup Phytoplasma Strain Associated with Pisum sativum and Parthenium hysterophorus L
Source: Plants (Basel). 2023 Feb 16;12(4):891. doi: 10.3390/plants12040891 (PMC9962045; doi:10.3390/plants12040891)
Supplement: Supplementary file 1 [file plants-12-00891-s001.zip › plants-2120079-supplementary.pdf]

Supplementary data

Yi-Ching Chiu

Plants

Detection, Identification and Molecular Characterization of the 16SrII-V Subgroup Phytoplasma Strain Associated with *Pisum sativum* and *Parthenium hysterophorus* L.

Yi-Ching Chiu [1,2,†], Pei-Qing Liao [1,†], Helen Mae Mejia [1], Ya-Chien Lee [1], Yuh-Kun Chen [3] and Jun-Yi Yang [1,2,4,5,*]

[1] Institute of Biochemistry, National Chung Hsing University, Taichung 402, Taiwan
[2] PhD Program in Microbial Genomics, National Chung Hsing University and Academia Sinica, Taichung 402, Taiwan
[3] Department of Plant Pathology, National Chung Hsing University, Taichung 402, Taiwan;
[4] Institute of Biotechnology, National Chung Hsing University, Taichung 402, Taiwan
[5] Advanced Plant Biotechnology Center, National Chung Hsing University, Taichung 402, Taiwan
† These authors contributed equally to this work.

Figures and Tables

A

B

C

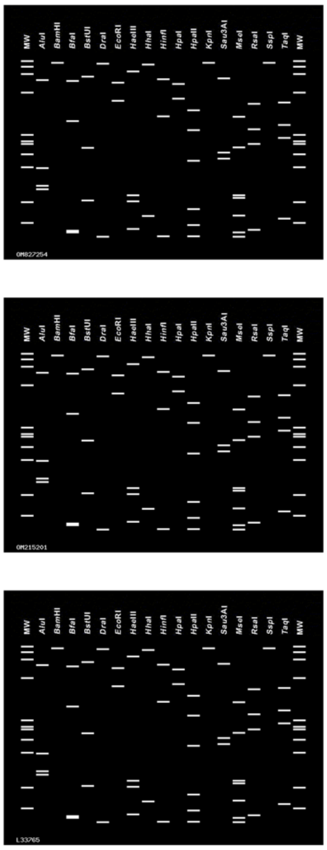

D

x: L33765
y: OM827254

Enzyme	N_x	N_y	N_{xy}
AluI(AGCT)	4	4	4
BamHI(GGATCC)	1	1	1
BfaI(CTAG)	4	4	4
BstUI(CGCG)	3	3	3
DraI(TTTAAA)	2	2	2
EcoRI(GAATTC)	2	2	2
HaeIII(GGCC)	4	4	4
HhaI(GCGC)	2	2	2
HinfI(GANTC)	3	3	3
HpaI(GTTAAC)	2	2	2
HpaII(CCGG)	6	6	6
KpnI(GGTACC)	1	1	1
MseI(TTAA)	7	7	7
RsaI(GTAC)	4	4	4
Sau3AI(GATC)	3	3	3
SspI(AATATT)	1	1	1
TaqI(TCGA)	4	4	4

x: L33765
y: OM215201

Enzyme	N_x	N_y	N_{xy}
AluI(AGCT)	4	4	4
BamHI(GGATCC)	1	1	1
BfaI(CTAG)	4	4	4
BstUI(CGCG)	3	3	3
DraI(TTTAAA)	2	2	2
EcoRI(GAATTC)	2	2	2
HaeIII(GGCC)	4	4	4
HhaI(GCGC)	2	2	2
HinfI(GANTC)	3	3	3
HpaI(GTTAAC)	2	2	2
HpaII(CCGG)	6	6	6
KpnI(GGTACC)	1	1	1
MseI(TTAA)	7	7	7
RsaI(GTAC)	4	4	4
Sau3AI(GATC)	3	3	3
SspI(AATATT)	1	1	1
TaqI(TCGA)	4	4	4

Supplementary Figure S1. The virtual RFLP pattern analysis by *i*PhyClassifier. *In silico* digestion of the 1.2 kb DNA fragment of the 16S rRNA gene of *Pisum sativum* witches' broom phytoplasma (accession No. OM827254) (**A**), *Parthenium hysterophorus* witches' broom phytoplasma (accession No. OM215201) (**B**), and peanut witches' broom phytoplasma (accession No. L33765) (**C**) were performed to generate the virtual RFLP patterns. **D**, The comparison details of virtual RFLP patterns of L33765 (x) and OM827254 (y) or OM215201 (y) are presented. N_x and N_y are the total numbers of DNA fragments resulting from enzyme digestions in x and y, respectively. N_{xy} is the number of DNA fragments shared by x and y.

Supplementary Table S1 Primer sequences

**Name**	**Sequence**	
P1	5' AAGAGTTTGATCCTGGCTCAGGATT 3'	For
P7	5' CGTCCTTCATCGGCTCTT 3'	Rev
R16F2n	5' GAAACGACTGCTAAGACTGG 3'	For
R16R2	5' TGACGGGCGGTGTGTACAAACCCCG 3'	Rev
Imp F	5' ATCG GGATCC ATAAAAACATTAACAACTAAAGAT 3' [a]	EPWB IMP (-TM) For
Imp R	5' CTAG CTCGAG TTATGATTGTAATTTTAAGTCTGT 3' [b]	EPWB IMP (-TM) Rev
PHYL1 F	5' CCAAAATATGTTAACTCGTGC 3'	Phytoplasma PHYL1 for
PHYL1 R	5' TGTTCATATTATGAAAACTCC 3'	Phytoplasma PHYL1 rev
SAP11 F	5' TTGAAACCAACCAACTTATAG 3'	Phytoplasma SAP11 for
SAP11 R	5' ACTG CATATG ATCCCGAATGAGGAATTCATC 3' [c]	Phytoplasma SAP11 rev

[a] Underline indicates the restriction enzyme cut site for BamHI.

[b] Underline indicates the restriction enzyme cut site for XhoI.

[c] Underline indicates the restriction enzyme cut site for NdeI.
